# Supplementary material for: Multimodal biometric identification: leveraging convolutional neural network (CNN) architectures and fusion techniques with fingerprint and finger vein data
Source: PeerJ Comput Sci. 2024 Oct 31;10:e2440. doi: 10.7717/peerj-cs.2440 (PMC11623012; doi:10.7717/peerj-cs.2440)
Supplement: Supplemental Information 3 [file peerj-cs-10-2440-s003.docx]

The table below provides a comprehensive overview of the computing infrastructure and software environment used to ensure the reproducibility of the study's results.

| **Component** | **Details** |
| --- | --- |
| **Operating System** | Ubuntu 20.04 LTS |
| **CPU** | Intel Core i9-10900K, 10 cores, 20 threads, base clock 3.7 GHz, max turbo frequency 5.3 GHz |
| **GPU** | NVIDIA GeForce RTX 3090, 24GB GDDR6X VRAM, CUDA cores: 10496, boost clock 1.70 GHz |
| **RAM** | 64GB DDR4, 3200 MHz |
| **Storage** | 2TB NVMe SSD, read speed up to 3,500 MB/s, write speed up to 3,300 MB/s |
| **Motherboard** | ASUS ROG Strix Z490-E Gaming |
| **Power Supply** | 850W 80 Plus Gold |
| **Cooling System** | Corsair Hydro Series H150i Pro RGB Liquid CPU Cooler |
| **Deep Learning Frameworks** | TensorFlow 2.4.1, PyTorch 1.8.1 |
| **Image Processing Libraries** | OpenCV 4.5.1, scikit-image 0.18.1 |
| **Data Handling Libraries** | Pandas 1.2.3, NumPy 1.20.1 |
| **Dataset** | NUPT-FPV (finger veins and fingerprint images) (https://github.com/REN382333467/NUPT-FPV) |
| **CNN Architectures** | ResNet (ResNet-50), VGGNet (VGG-16), DenseNet (DenseNet-121) |
| **Fusion Strategies** | Early Fusion, Late Fusion, Score-level Fusion |
| **Enhancement Method** | Contrast Limited Adaptive Histogram Equalization (CLAHE) |
| **Evaluation Metrics** | Accuracy, Equal Error Rate (EER), Receiver Operating Characteristic (ROC) curves |
| **Development Environment** | Jupyter Notebook, Python 3.8 |
| **Version Control** | Git (GitHub repository for version control and collaboration) |
| **Documentation Tools** | Sphinx, Markdown |
| **Virtualization/Containers** | Docker (Docker images for consistent environment setup) |
| **Code Libraries and Utilities** | Scikit-learn 0.24.1 (for additional machine learning utilities), Matplotlib 3.3.4 (for plotting and visualization) |
| **Data Preprocessing Techniques** | Normalization, Augmentation (rotation, scaling, translation) |
| **Training Parameters** | Batch Size: 32, Learning Rate: 0.001, Optimizer: Adam, Epochs: 50 |
| **Hyperparameter Tuning** | Grid Search, Random Search |
| **Validation Techniques** | Cross-Validation (K-Fold, k=5) |
| **Logging and Monitoring** | TensorBoard for tracking training progress and performance metrics |
| **Backup and Recovery** | Regular snapshots of the environment and data, automated backups using rsync |
