# Supplementary material for: Multimodal biometric identification: leveraging convolutional neural network (CNN) architectures and fusion techniques with fingerprint and finger vein data
Source: PeerJ Comput Sci. 2024 Oct 31;10:e2440. doi: 10.7717/peerj-cs.2440 (PMC11623012; doi:10.7717/peerj-cs.2440)
Supplement: Supplemental Information 4 [file peerj-cs-10-2440-s004.docx]

**Justification for Model Type Used**

1. ResNet (ResNet-50): It has been chosen due to the characteristic of this model to resolve the vanishing gradient problem with deep networks. ResNet-50 is based on residual blocks, which simplifies the process of training very deep networks. It enables to extract the smallest details from biometric images.
2. VGGNet (VGG-16): The model is based on a deep, but uniform architecture, which includes merely differently sized stacked convolutional layers. VGGNet is relatively simple, but effective at image classification . It provides a good performance in the process of feature extraction.
3. DenseNet (DenseNet-121): The model has been employed since it is based on densely connected architecture. It supports maximum information flow between the units in any two close network layers. This type of connectivity is important for the dense patterns of extraction and helps to avoid a vanishing gradient problem.

**Justification for Using Contrast Limited Adaptive Histogram Equalization (CLAHE) :**

CLAHE is used to enhance the contrast of fingerprint images and outline the vein patterns in finger vein images. As a preprocessing step, it is essential to facilitate the visibility of fine details in the biometric trait images. Notably, clear visibility of features helps to improve the feature extraction process by CNN models. The use of CLAHE is preferred over the standard histogram equalization as the latter tends to over-amplify noise in relatively uniform regions of an image. CLAHE works by dividing the image into small regions or tiles and applying equalization to each of these tiles. These regions are then combined using bilinear interpolation to eliminate artificially induced visible boundaries. CLAHE also applies a limit to the amount of amplification or the equalization. Notably, the process used a threshold or the clip limit to prevent over-amplification of noise.

**Evaluation Method**

1. Early Fusion: It combines raw fingerprint and finger vein at the input layer of CNN. This strategy exploits the fact that the information is integrated at the earliest level, and the network can learn joint representation.
2. Late Fusion: Merges the features extracted individually by the three CNN models following the processing of the images. This allows each model to learn the modality-specific features before combining them for decision-making.
3. Score Level Fusion: Aggregates the matched scores from each modality using a weighted approach, taking advantage of the complementary information provided by each biometric trait.

**Evaluation Method for CLAHE**

- **Visual Inspection:** The enhanced images are visually inspected to ensure that the contrast of the fingerprint ridges and finger vein patterns are significantly improved without introducing artifacts.
- **Feature Extraction Quality:** The quality of features extracted by the CNN models from CLAHE-processed images is compared to those extracted from non-processed images. The improvement in feature extraction is assessed by the performance of the CNN models in subsequent tasks (e.g., accuracy, EER).

**Selection Method**

1. Hyperparameter Tuning:

- Grid Search: It searches the best combination between the hyperparameter of the model, sweeping the grid and collecting the results of these sweeps to select the best ones.
- Random Search: The way it works is that we define a range of hyperparameters and pick the hyperparameters of the model randomly from these defined, values which is applicable in high dimensional spaces.

1. Cross-Validation:
   - K-Fold Cross-Validation (k=5): This is where you divide the dataset into k-folds, each fold result to a model with its performance metrics being recorded with the remaining folds considered as a training set. This process is repeated k-times using a different i-th fold as testing set with the final performance metric being the average of all the performance metrics.

**Selection Method and Validation for CLAHE**

1. **Tile Size:** The size of the tiles for CLAHE is selected based on experimentation. Typically, smaller tiles provide finer contrast enhancements, while larger tiles offer more global adjustments.
2. **Clip Limit:** The clip limit, which controls the contrast enhancement, is tuned to balance enhancement and noise suppression.

The performance of models using CLAHE-enhanced images is validated using the same cross-validation techniques (e.g., K-Fold Cross-Validation) employed for the overall model assessment.

**Assessment Metrics (Justification)**

1. **Accuracy Improvement:** The increase in model accuracy when using CLAHE-enhanced images compared to raw images. This metric indicates the effectiveness of CLAHE in improving the discriminative power of features extracted by the CNN models.
2. **EER Reduction:** The decrease in Equal Error Rate (EER) when using CLAHE-enhanced images. A lower EER signifies better balance between false acceptance and false rejection rates, demonstrating the utility of CLAHE in enhancing security and usability.
3. **AUC Increase:** Improvement in the area under the ROC curve (AUC) with CLAHE-enhanced images. A higher AUC indicates better overall model performance across various threshold settings, showing that CLAHE contributes to more robust and reliable biometric identification.

These metrics validate the effectiveness of the CLAHE technique in preprocessing biometric images, enhancing the contrast and visibility of critical features, and ultimately improving the performance of the CNN models in multimodal biometrics identification.
