# Supplementary material for: Multimodal biometric identification: leveraging convolutional neural network (CNN) architectures and fusion techniques with fingerprint and finger vein data
Source: PeerJ Comput Sci. 2024 Oct 31;10:e2440. doi: 10.7717/peerj-cs.2440 (PMC11623012; doi:10.7717/peerj-cs.2440)
Supplement: Supplemental Information 5 [file peerj-cs-10-2440-s005.docx]

1. Dataset Limitations:
   - Large but Not Extensive: The NUPT-FPV dataset, despite being large and extensive, may fail to cover all possible variations of fingerprint and finger vein images as they can take in real world. Thus, the scope may not guarantee high generalization.
   - Challenge of Diversity: It may originate from limited diversity of such aspects as age or ethnicity and the environment in terms of the equipment settings or lighting. The unexpected occurrences may be experienced in reality.
2. Model Complexity:
   - High Computational Density: Deep CNN architectures including ResNet, VGGNet, and DenseNet, the model described herein, are computationally expensive due to training and inference.
   - Training Time: The necessity to train a deep model along with multiple fusion imposes the risks of model overfitting. At the same time, integrating one specification or change takes too much time for rapid commercial deployment.
3. Fusion Techniques:
   - Implementation Variability: The implementation of a particular fusion type, such as early, late, or score-level, may be different. At the same time, the performance may heavily rely on correct weight specification and validation. Thus, they can be quite sensitive.
   - Integration Complexity: Combining multiple biometric modalities and fusion techniques increases the complexity of the system, potentially leading to integration and maintenance challenges.
4. Preprocessing Limitations:
   - Enhanced Artifacts: While CLAHE has an array of benefts including improved contrast and features visibility, it should not be the only solution. In fact, over-enhancement and under-enhancement create artifacts. Thus, it may not be suitable for each type of the biometric images.
   - Parameter Dependency: Parameter choices may differ for every biometric image.
